# Diversity Profiling of Grapevine Microbial Endosphere and Antagonistic Potential of Endophytic *Pseudomonas* Against Grapevine Trunk Diseases

**DOI:** 10.3389/fmicb.2020.00477

**Published:** 2020-03-26

**Authors:** Jennifer Millera Niem, Regina Billones-Baaijens, Benjamin Stodart, Sandra Savocchia

**Affiliations:** ^1^National Wine and Grape Industry Centre, Charles Sturt University, Wagga Wagga, NSW, Australia; ^2^School of Agricultural and Wine Sciences, Charles Sturt University, Wagga Wagga, NSW, Australia; ^3^Graham Centre for Agricultural Innovation (Charles Sturt University and NSW Department of Primary Industries), School of Agricultural and Wine Sciences, Charles Sturt University, Wagga Wagga, NSW, Australia

**Keywords:** *Pseudomonas poae*, microbiome, endophyte, biocontrol, Botryosphaeria dieback, Eutypa dieback, Esca/Petri disease

## Abstract

Grapevine trunk diseases (GTDs) are a serious problem of grapevines worldwide. The microbiota of the grapevine endosphere comprises prokaryotic and eukaryotic endophytes, which may form varied relationships with the host plant from symbiotic to pathogenic. To explore the interaction between grapevine endophytic bacteria and GTDs, the endomicrobiome associated with grapevine wood was characterized using next-generation Illumina sequencing. Wood samples were collected from grapevine trunks with and without external symptoms of GTD (cankers) from two vineyards in the Hunter Valley and Hilltops, NSW, Australia and metagenomic characterization of the endophytic community was conducted using the 16S rRNA gene (341F/806R) and ITS (1F/2R) sequences. Among the important GTD pathogens, *Phaeomoniella, Phaeoacremonium, Diplodia* and *Cryptovalsa* species were found to be abundant in both symptomatic and asymptomatic grapevines from both vineyards. *Eutypa lata* and *Neofusicoccum parvum*, two important GTD pathogens, were detected in low numbers in Hilltops and the Hunter Valley, respectively. Interestingly, *Pseudomonas* dominated the bacterial community in canker-free grapevine tissues in both locations, comprising 56–74% of the total bacterial population. In contrast, the *Pseudomonas* population in grapevines with cankers was significantly lower, representing 29 and 2% of the bacterial community in Hilltops and the Hunter Valley, respectively. The presence of *Pseudomonas* in healthy grapevine tissues indicates its ability to colonize and survive in the grapevine. The potential of *Pseudomonas* spp. as biocontrol agents against GTD pathogens was also explored. Dual culture tests with isolated fluorescent *Pseudomonas* against mycelial discs of nine Botryosphaeria dieback, three Eutypa dieback, and two Esca/Petri disease pathogens, revealed antagonistic activity for 10 *Pseudomonas* strains. These results suggest the potential of *Pseudomonas* species from grapevine wood to be used as biocontrol agents to manage certain GTD pathogens.

## Introduction

Grapevine trunk diseases (GTDs) are economically important in all grape growing regions and significantly impact grape production and the wine industry worldwide. These diseases are a major threat to the Australian wine industry and affect the long-term sustainability and productivity of many vineyards. Esca, Petri and black foot diseases, and Eutypa and Botryosphaeria diebacks are the major constraints in Australian vineyards potentially causing considerable economic loss to the industry (Wicks and Davies, 1999; Edwards and Pascoe, 2004; Whitelaw-Weckert et al., 2013; Billones-Baaijens and Savocchia, 2019). Once established, these diseases are often difficult to control and can cause establishment problems for young vines and the decline of established vines (Gramaje et al., 2018). The general symptoms of these diseases are wood necrosis characterized by brown streaking or cankers, foliar discoloration and drying. Symptoms of GTDs usually take several years to develop, making early detection difficult. Pruning wounds are the main point of entry for fungal spores (Slippers and Wingfield, 2007; Gramaje et al., 2018). Protection of pruning wounds is deemed essential to reduce infections from GTD pathogens (Mondello et al., 2018). The causal organisms grow, decay the wood and eventually kill the vines.

Botryosphaeria canker and dieback are caused by species of fungi in the *Botryosphaeriaceae* family. Many species associated with Botryosphaeria canker were grouped within the genus *Botryosphaeria*, hence, the disease is commonly referred to as Botryosphaeria dieback. However, current reclassification now includes the genera *Fusicoccum, Neofusicoccum, Diplodia, Lasiodiplodia, Dothiorella, Spencermartinsia*, and *Sphaeropsis* (Úrbez-Torres et al., 2015). Eutypa dieback is caused by *Eutypa lata* and other *Diatrypaceae* species including *Anthostoma, Cryptosphaeria, Cryptovalsa, Diatrype, Diatrypella*, and *Eutypella* (Trouillas et al., 2011; Luque et al., 2012). The etiology of Esca disease is still not fully understood. Although a number of unrelated microorganisms have been isolated from Esca-infected vines, their role and interaction with each other are not clear (Gramaje et al., 2018). However, *Phaeomoniella chlamydospora* and/or species of *Phaeoacremonium* are commonly associated with the disease. Although some authors claimed several basidiomycetous species belonging to the genera *Fomitiporia, Phellinus, Stereum, Fomitiporella, Inonotus*, and *Inocutis* to be requisites for the development of classic Esca symptoms (Cloete et al., 2015; Gramaje et al., 2018), others suggested that young esca is induced by *Phaeomoniella chlamydospora* alone (Edwards et al., 2001). Petri disease or black goo decline is primarily associated with *P. chlamydospora* (Gubler et al., 2015) although several species of *Phaeoacremonium*, *Pleurostoma*, and *Cadophora* have recently been linked to the disease (Gramaje et al., 2018).

Currently there are no reliable curative control measures against GTDs and there are no means, chemical or other, to eradicate the organisms once they become established within a vine. As eradication is problematic, disease control is mainly though disease prevention and mitigation (Úrbez-Torres, 2011). Management of GTDs is mainly though employment of cultural practices that include vineyard sanitation to reduce the inoculum density, remedial surgery wherein the infected parts of the vines are excised, and appropriate timing of pruning and training of vines (Gramaje et al., 2018). In Spain, anecdotal evidence has been presented for the use of copper nails placed in the scion parts of grapevine trunks, which slowed the progress of wood rot in infected plants, but this is yet to be scientifically validated (Mondello et al., 2018). Fungicides such as the benzimidazole carbamate group, tebuconazole, flusilazole, pyrimethanil, pyraclostrobin, and fluazinam are also used as pruning wound protection against Botryosphaeria and Eutypa dieback pathogens (Gramaje et al., 2018). For Esca and Petri disease pathogens, thiophanate-methyl and boron are found to be more effective pruning wound protectants (Rolshausen et al., 2010). However, the downside with fungicide usage is that some fungicides may only provide short-term protection. This may be problematic, since pruning wounds remain susceptible to *E. lata* for 2–4 weeks and up to 16 weeks to species of *Botryosphaeriaceae* (Munkvold and Marois, 1995; Úrbez-Torres and Gubler, 2011; Ayres et al., 2016). Furthermore, there are only limited fungicides that are commercially available to manage GTDs.

Biological control agents (BCAs) may be a potential strategy to supplement chemical methods. In recent years, the use of endophytic BCAs in the management of plant disease has gained popularity as an alternative to chemical application, driven in part by concerns for human and environmental safety with the use of fungicides (Chen et al., 1995; Ryan et al., 2008; Cabanás et al., 2014; Rybakova et al., 2015; Aydi Ben Abdallah et al., 2016; Hong and Park, 2016). Endophytes are microorganisms that spend at least parts of their life cycle inside the plant (Hardoim et al., 2015). They are ubiquitous in their host plant and may reside latently or actively colonize plant tissues (Hallmann et al., 1997). Most endophytes are commensals which reside within the plant tissues and live on metabolites produced by their host, often without any known effect on the plant (Hardoim et al., 2015; Brader et al., 2017). Another group of endophytes has mutualistic relationship with plants (Hardoim et al., 2015; Brader et al., 2017) and provide benefits to their host though promotion of plant growth, biocontrol of plant pathogens, enhancement of plant nitrogen fixation and phosphate solubilization (Hallmann et al., 1997; Iniguez et al., 2004; Ryan et al., 2008; Rybakova et al., 2015). Some endophytes may also exhibit pathogenicity when conditions become favorable (Hardoim et al., 2015; Brader et al., 2017).

Limited studies have been conducted on the grapevine endophytic microbial community, with most research focusing on the microbial composition of the grapevine phyllosphere (Leveau and Tech, 2011; Bokulich et al., 2013; Perazzolli et al., 2014; Pinto et al., 2014; Portillo et al., 2016). Moreover, most of the studies conducted on grapevine microbiota are focused on bacterial endophytes (Bell et al., 1995; Bulgari et al., 2009, 2011; West et al., 2010; Compant et al., 2011; Campisano et al., 2014, 2015; Andreolli et al., 2016) and much less on endophytic fungi. Deyett et al. (2017) conducted a comprehensive study of both the bacterial and fungal assemblages in the grapevine endosphere of a Californian vineyard and found a negative correlation between the Pierce’s disease pathogen, *Xylella fastidiosa* and the endophytic bacteria *Pseudomonas fluorescens* and *Achomobacter xylosoxidans*. In Spain, Elena et al. (2018) characterized the fungal and bacterial composition and diversity in GTD asymptomatic and symptomatic vines and found that there were no differences in the fungal communities between the healthy and diseased vines, while differences in the bacterial composition in non-necrotic and necrotic tissues were evident. The lone attempt to study the grapevine microbial profile in Australia was conducted by West et al. (2010), characterizing the diversity of culturable and non-culturable bacterial endophytes in grapevines. However, microbial distribution patterns may differ within and between geographical locations due to differences in environmental conditions (Bokulich et al., 2013). In Australia, Eutypa dieback and Botryosphaeria dieback are two of the most important GTDs (Scholefield and Morison, 2010; Billones-Baaijens and Savocchia, 2019). To our knowledge, studies on the use of grapevine endophytes to control Eutypa and Botryosphaeria diebacks in grapevines have not been conducted. There is a need therefore to investigate the potential of grapevine endophytic organisms as BCAs for dieback diseases, providing environmentally sustainable solutions to a major problem in the Australian wine industry. This study was conducted to identify the microbiome inhabiting the inner grapevine tissues and to examine the interaction between grapevine endophytic bacteria and GTD pathogens. Furthermore, the biocontrol potential of the most dominant endophytic bacterium against GTD pathogens was explored. Beneficial microorganisms that occur naturally within plant tissues have potential as effective BCAs as they are adapted to the plant host they were originally isolated from and may have greater potential to be sustained within the host.

## Materials and Methods

### Plant Sample Collection

Samples were collected during the spring of 2016 from commercial vineyards in Hilltops and the Hunter Valley, which are the major grape-growing regions in New South Wales, Australia. These vineyards were selected as GTDs were previously reported to occur in these areas (Baaijens, personal communication). Vineyard surveys identified *Botryosphaeriaceae* and *Diatrypaceae* species as being present in these regions (Pitt et al., 2010; Qiu et al., 2011; Trouillas et al., 2011) while Esca/Petri disease pathogens were found in the Hunter Valley and inland NSW. For the Hunter Valley, 10 asymptomatic (no visible cankers) and 10 symptomatic (exhibiting cankers) Verdelho vines (18 years old) were sampled. For Hilltops, 20 years old Shiraz vines that had previously undergone remedial surgery (Savocchia et al., 2014) were sampled. These vines (10 each) exhibited internal necroses or were necroses-free when trunks were cut during surgery. The selected vines were debarked with a surface-sterilized knife and wood shavings (∼1 g) were obtained using an 8 mm auger bit driven by a CDL-018 cordless drill drive (Ozito Industries Pty. Ltd., Victoria, Australia). The drill bit was sprayed with ethanol for each vine sampled to prevent cross contamination. Wood shavings were placed in sterile plastic containers and transported to the laboratory on ice. One half of each sample was stored at 4°C prior to isolation of microorganisms, while the remaining half was stored at −80°C prior to DNA extraction.

### DNA Extraction From Grapevine Wood Samples

DNA was extracted from 100 mg of ground wood shavings from both asymptomatic and symptomatic inner grapevine tissues following the methods of Pouzoulet et al. (2013) with slight modifications. Frozen samples were lyophilized for 24 h in a freeze drier (John Morris Scientific, United States) and ground at 20 Hz for 2 min in a TissueLyser II (Qiagen, United States) within 10 ml stainless-steel grinding jars, containing 20 mm stainless steel balls (Qiagen, United States). Total DNA was extracted from pulverized freeze-dried wood samples using the CTAB extraction buffer of Doyle and Doyle (1987). Pulverized wood samples were incubated in CTAB buffer at 65°C for 1 h, and then extracted using chloroform/isoamyl alcohol (24:1) (Sigma-Aldrich, United States) on ice for 5 min. The mixture was then centrifuged at 4°C for 10 min at 2300 × g. The lysate was then transferred to a QIAshredder spin column provided in the DNeasy Plant Mini Kit (Qiagen, United States). Following this procedure, the subsequent steps indicated in the DNeasy Plant Mini Kit were followed. DNA samples were eluted to a final volume of 100 μl using AE buffer (Qiagen, United States), and stored at −20°C. DNA concentration was determined using a Quantus Fluorometer (Promega, United States). The quality of DNA was further verified though gel electrophoresis and DNA samples (∼70 ng) were used for microbial diversity profiling analysis. Raw sequence reads are available from the NCBI’s Sequence Read Archive (Bioproject PRJNA605898).

### Sequencing of the 16S and ITS rRNA Genes

PCR amplification and sequencing was performed at the Australian Genome Research Facility (AGRF, Sydney, NSW, Australia). Amplicons for both 16S and ITS were generated using the primers and conditions outlined in Table 1. Thermocycling was completed with an Applied Biosystem 384 Veriti and using AmpliTaq Gold 360 (Life Technologies, Australia). Illumina indexing of the amplicons was achieved in a second PCR utilizing TaKaRa Taq DNA Polymerase (Clontech). Indexed amplicon libraries were quantified by fluorometry (Promega Quantifluor) and normalized. An equimolar pool was created and adjusted to 5 nM for sequencing on an Illumina MiSeq (San Diego, CA, United States) with a V3, 600 cycle kit (2 × 300 base pair, paired-end reads).

**TABLE 1:** Primers and PCR conditions for 16S and ITS amplicons.

Target	Cycle	Initial	Disassociate	Anneal	Extension	Finish
ITS1F–ITS2	35	95°C for 7 min	94°C for 30 s	55°C for 45 s	72°C for 60 s	72°C for 7 min
16S: V3–V4	29	95°C for 7 min	94°C for 30 s	50°C for 60 s	72°C for 60 s	72°C for 7 min

### Isolation of Fluorescent *Pseudomonas* From Inner Grapevine Tissues

As the next generation sequencing data for microbial profiling revealed *Pseudomonas* to be the predominant endophytic bacterium in grapevine tissues, these were targeted for isolation from the collected grapevine wood samples. Approximately 0.5 g of wood shavings per sample were suspended in 6 ml of 3 × Ringer’s solution (Surette et al., 2003). Samples were homogenized by incubating in a rotary shaker for 2 h at 240 rpm under ambient temperature. A dilution series of up to 10^–6^ was prepared using 3 × Ringer’s solution and plated on nutrient agar (NA, Oxoid Ltd., Hampshire, United Kingdom). Well separated and distinct bacterial colonies were subcultured onto King’s B medium (King et al., 1954). Additional bacterial strains were further isolated from pruning stubs collected from Cabernet Sauvignon vines from the vineyard of Charles Sturt University, NSW, Australia.

The pure cultures obtained from the vineyards were observed for fluorescence on King’s B media, under UV light at 365 nm after 3 days of incubation at 25°C in the dark. Fluorescent bacterial strains from single colonies were stored in 20% glycerol at −80°C.

### *In vitro* Screening for Bacterial Antagonists Against GTD Pathogens

#### Bacterial Strains

A total of 57 fluorescent bacterial strains from inner grapevine wood tissues were tested for their antagonistic activity against the GTD pathogens: (a) *Diplodia seriata*; (b) *Neofusicoccum parvum*; (c) *Neofusicoccum luteum;* and (d) *Eutypa lata*, which are the most prevalent and virulent GTD pathogens in Australia. From this initial screen, 10 strains (Table 2) demonstrating inhibition of the GTD pathogens were further tested for their antagonistic activity against 14 GTD pathogens that are associated with Botryosphaeria dieback, Eutypa dieback, and Esca/Petri disease (Table 3).

**TABLE 2:** Fluorescent bacterial strains with antagonistic activity against GTD pathogens *Diplodia seriata*, *Neofusicoccum parvum*, *N. luteum*, and *Eutypa lata.*

Bacterial strains	Identification based on 16S rRNA gene	GenBank accession no.
BCA11	*Pseudomonas poae*	MN480480
BCA12	*P. moraviensis*	MN480481
BCA13	*P. poae*	MN480482
BCA14	*P. poae*	MN480483
BCA15	*P. poae*	MN480484
BCA16	*P. poae*	MN480485
BCA17	*P. poae*	MN480486
BCA18	*P. poae*	MN480487
BCA19	*P. poae*	MN480488
BCA20	*P. poae*	MN480489

**TABLE 3:** Fungal strains used for *in vitro* assays to test the antagonistic activity of *Pseudomonas* strains.

Fungal species	Isolate	Herbarium accession^a^	References
*Botryosphaeria dothidea*	BMV14	DAR79239	Pitt et al., 2010
*Diplodia mutila*	FF18	DAR79137	Pitt et al., 2010
*D. seriata*	A142a	DAR79990	Qiu et al., 2011
*Dothiorella vidmadera*	L5	DAR78993	Pitt et al., 2013
*Lasiodiplodia theobromae*	W200	DAR81024	Wunderlich et al., 2011
*Neofusicoccum australe*	SDW4	DAR79505	Pitt et al., 2010
*N. luteum*	BB175-2	DAR81016	Wunderlich et al., 2011
*N. parvum*	B22a	DAR80004	Qiu et al., 2011
*Spencermartinsia viticola*	L19	DAR78870	Pitt et al., 2010
*Cryptovalsa ampelina*	KC6		Trouillas et al., 2011
*Eutypa lata*	WB052		Billones-Baaijens et al., 2018
*Eutypella citricola*	WA06FH		Trouillas et al., 2011
*Phaeoacremonium minimum*	SMA 056		Billones-Baaijens et al., 2018
*Phaeomoniella chlamydospora*	SMA 006		Billones-Baaijens et al., 2018

*^a^DAR – Agricultural Scientific Collections Unit, NSW DPI, Orange, Australia.*

#### Fungal Strains

Fourteen fungal strains associated with GTDs were obtained from the National Wine and Grape Industry Centre (Charles Sturt University, Wagga Wagga, NSW, Australia) culture collection (Table 2). Each of the selected strains had been stored on agar discs in 20% glycerol at −80°C prior to testing. All *Botryosphaeriaceae* strains were associated with Botryosphaeria dieback symptoms in Australian vineyards (Pitt et al., 2010; Qiu et al., 2011; Wunderlich et al., 2011), while all *Diatrypaceae* strains were associated with Eutypa dieback symptoms (Trouillas et al., 2011). The *Phaeoacremonium minimum* and *Phaeomoniella chlamydospora* strains were obtained from the culture collection of the South Australian Research and Development Institute (SARDI, Adelaide, Australia). All strains were previously identified to species level by DNA sequencing of the ITS region of the rRNA and β-tubulin genes (Pitt et al., 2010, 2013; Qiu et al., 2011; Wunderlich et al., 2011; Billones-Baaijens et al., 2018).

#### Dual Culture Assays

To study the ability of fluorescent *Pseudomonas* spp. (Table 1) to inhibit growth of the GTD pathogens (Table 2), dual plating assays were performed. For each pathogen, mycelial plugs 6 mm in diameter were placed on opposite sides of potato dextrose agar (PDA, Difco Laboratories, Maryland, United States) plates. A 10 μl *Pseudomonas* suspension (1 × 10^8^ CFU/ml), grown for 24 h in nutrient broth (NB, Oxoid Ltd., Hampshire, United Kingdom), was then pipetted in the centre of the PDA plates. Petri plates containing mycelial plugs of the pathogens only served as controls. Three replicate plates were used for each bacteria-fungal pathogen treatment combination, and incubated at 25°C under continuous darkness. Mycelial growth of the fungi was measured after 7, 10, and 30 days to evaluate inhibition of the Botryosphaeria dieback, Eutypa dieback and Esca/Petri disease pathogens, respectively, by the antagonistic *Pseudomonas* species. Average fungal growth was determined by measuring the perpendicular diameter of the mycelial growth of the respective GTD pathogens. Percentage mycelial growth inhibition was calculated using the formula:

$$\mathrm{Percentage}\,\mathrm{mycelial}\,\mathrm{growth}\,\mathrm{inhibition}=\frac{\left(C-T\right)}{C}\times 100$$

Where C = colony diameter (mm) of the control

T = colony diameter (mm) of the test plate

In the case of *Ph. minimum*, the bioassays were conducted using a spore suspension of the fungi in the well dual culture. *Ph. minimum* readily produces abundant spores in PDA and spores were harvested from a 7 days old culture of the fungus. Spore concentration was adjusted to 1 × 10^6^ spores/ml with a hemocytometer and 100 μl of the spore suspension was spread onto PDA plates. Four equidistant holes were created in the media using a flame-sterilized cork borer (4 mm) and 10 μl of the bacterial suspension was pipetted into each well. For the control plates, sterile distilled water was pipetted into the wells. Three replicate plates per treatment combination were used. The diameter of the zone of inhibition was measured around each well after 7 days of incubation under the conditions described above.

#### Identification of Selected Bacterial Strains by 16S rRNA Gene Sequencing

The 10 fluorescent bacteria that significantly inhibited the mycelial growth of the GTD fungal pathogens and 11 additional non-antagonistic fluorescent bacterial strains were selected for molecular identification. The bacterial growth conditions and DNA extraction method were as described by Niem et al. (2019), using the Gentra Puregene Bacterial DNA Extraction Kit (Qiagen, United States) following the manufacturer’s instructions.

The bacterial species were identified though PCR amplification of 16S rRNA gene using the universal primers 8F and 1492 R (I) (Turner et al., 1999). PCR amplification was performed with 1 μl of genomic DNA template in a 25 μl reaction mixture containing 1× PCR buffer (Bioline, United Kingdom), 1.25 U of My Taq DNA polymerase (Bioline, United Kingdom) and 0.4 μM of each primer. PCR was performed with a thermal cycler (C100 Thermal Cycler, BIO-RAD Laboratories Pty. Ltd., United States) under the following conditions: initial denaturation for 3 min at 95°C; 35 denaturation cycles of 15 s at 95°C, annealing for 1 min at 56°C, extension for 1 min at 70°C; and a final extension of 8 min at 72°C. PCR products were purified with a FavorPrep Gel/PCR purification kit (Favorgen Biotech Corp, United States) and sequenced at the AGRF.

To identify the bacterial species, all DNA sequences and chromatographs were analyzed and trimmed using Chromas Lite Version 2.6.2 (Technelysium Pty. Ltd.) and DNAMAN Version 6.0.3.99 (Lynnon Biosoft) and compared with sequence data in GenBank^1^ using the Basic Local Alignment Search Tool (BLAST) (Altschul et al., 1990) to determine the most probable identity for the 21 isolates examined. For phylogenetic analysis, published reference sequences for the 16S rRNA gene were obtained from the NCBI database and aligned with the 21 generated sequences, by Clustal W within MEGA 7 (Kumar et al., 2016). The aligned sequences were tested for their phylogeny (Tamura et al., 2004) and a neighbor joining tree was generated using MEGA 7 to determine the relationships between the reference and unknown sequences (Saitou and Nei, 1987).

#### Data Analysis

Paired-ends reads were assembled by aligning the forward and reverse reads using PEAR version 0.9.5 (Zhang et al., 2014). Primers were identified and removed, with trimmed sequences being processed using Quantitative Insights into Microbial Ecology (QIIME 1.8) (Caporaso et al., 2010), USEARCH (version 7.1.1090) (Edgar, 2010; Edgar et al., 2011), and UPARSE (Edgar, 2013) software. Within USEARCH, sequences were quality filtered, full length duplicate sequences removed, and reads sorted by abundance. Singletons or unique reads in the data set were discarded. Sequences were clustered followed by chimera filtering using “rdp_gold” database as the reference. To obtain the number of reads in each OTU, reads were mapped back to OTUs with a minimum identity of 97%. Taxonomy was assigned by QIIME using the Greengenes database (version 13_8 Aug 2013) (DeSantis et al., 2006). In the case of ITS sequences, these were clustered followed by chimera filtering using the “Unite” database as reference. To obtain number of reads in each OTU, reads were mapped back to OTUs with a minimum identity of 97%. Taxonomy was assigned by QIIME using the Unite database (Unite Version7.1 2016-08-22) (Kõljalg et al., 2005). Unless mentioned, default settings were used for all procedures.

Microbial diversity profiling data were generated from nine and eight samples each from asymptomatic and symptomatic vines from Hilltops and the Hunter Valley, respectively. Only those taxa that represent more than 1% of the bacterial and fungal operational taxonomic units (OTUs) were included in the data set (Deyett et al., 2017). Data for 0.01–0.9% of the fungal OTUs were included as an inset to take into account fungal genera that were obscured by high relative abundance of the dominant genus. Chloroplast and mitochondrial sequences or OTUs with unidentified taxa were removed. Percent relative abundance of the bacterial and fungal taxa inhabiting the grape inner tissues was calculated. The compositional diversity of the fungal and bacterial endophytic communities were considered separately. Diversities were compared with alpha (Chao1 and Simpson) and beta (Bray-Curtis index) diversity statistics within the Marker Data Profiling module of MicrobiomeAnalyst (Dhariwal et al., 2017), implementing R version 3.6.1 (2019-07-05). Abundance data for bacteria and fungi were normalized by data transformation using centered log ratio, as recommended by Gloor et al. (2017). Differences in the Chao1 and Simpson index among factors (location and presence/absence of symptoms) were determined via ANOVA when considering all factors, or *T*-tests for factors individually. For the beta diversity, differences were visualized using non-metric multidimensional scaling (NMDS). Permutational multivariate analysis of variance (PERMANOVA) was used to test which factors best explained the NMDS.

For the dual culture assay, all data were analyzed within GenStat (18th edition). Since mycelial growth rate differed between Botryosphaeria dieback, Eutypa dieback and Esca/Petri disease pathogens, assessments for each pathogen group were done at different periods and analyzed separately. Percent mycelial diameter inhibition for each pathogen group was initially tested for homogeneity using Levene’s test at *P* ≤ 0.05. Effects of treatments were determined by two-way analysis of variance (ANOVA). Pairwise comparison of treatment means was conducted using least significant differences (LSD) at *P* ≤ 0.05.

## Results

### Microbial Profile of Grapevine Endosphere

A total of 48,461 bacterial 16S rRNA gene sequences and 1,567,896 fungal ITS sequences were generated after removing mitochondrial and chloroplast sequences. These sequences were assigned to 573 bacterial and 244 fungal operational taxonomic units (OTUs).

### Bacterial Community

**FIGURE 1:**
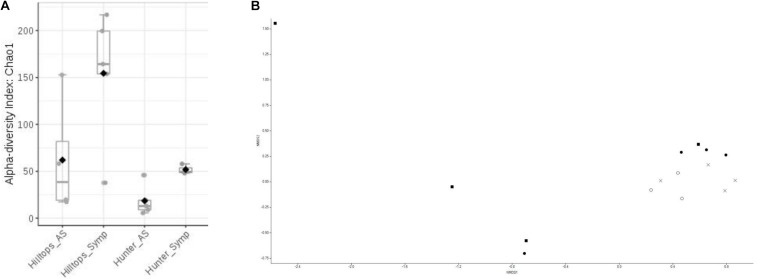
Diversity analysis of bacterial endophytic communities sampled from GTD symptomatic and asymptomatic vines, in the Hilltops and Hunter Valley regions. Abundance of OTUs was determined following the sequencing of the V3–V4 region of 16s rRNA genes: **(A)** Alpha-diversity measure using Chao1 at the OTU level, with each boxplot representing the diversity distribution of each factor (location and symptomology); and **(B)** Ordination based upon beta diversity analysis using the Bray Curtis index. Closed circles = Hilltops, asymptomatic; closed squares = Hilltops, symptomatic; X = hunter Valley, asymptomatic; O = Hunter Valley, symptomatic.

Alpha diversity analysis indicated that significant differences existed in terms of richness (Chao1) within communities of bacteria considering all factors (*F* = 7.13, *P* ≤ 0.05). Comparison between location suggested that the Hilltops region had significantly greater diversity in terms of OTU richness than the Hunter Valley (*t* = 3.07, *P* ≤ 0.05; Figure 1A). Taking this in to account the differences between asymptomatic and symptomatic samples from within each location were determined. For both Hilltops and the Hunter Valley asymptomatic samples were lower in terms of OTU richness than symptomatic, however, the differences were not significant. For richness and evenness (Simpson) there were no significant differences between factors.

In regards to the composition of bacterial communities, non-metric multidimensional scaling and PERMANOVA analysis indicated that communities could be separated when considering all factors (R-squared = 0.24, *P* ≤ 0.05; Figure 1B). This was attributable to compositional differences between location (R-squared = 0.14, *P* < 0.01) rather than expressed symptomology (R-squared = 0.07, *P* > 0.05).

**FIGURE 2:**
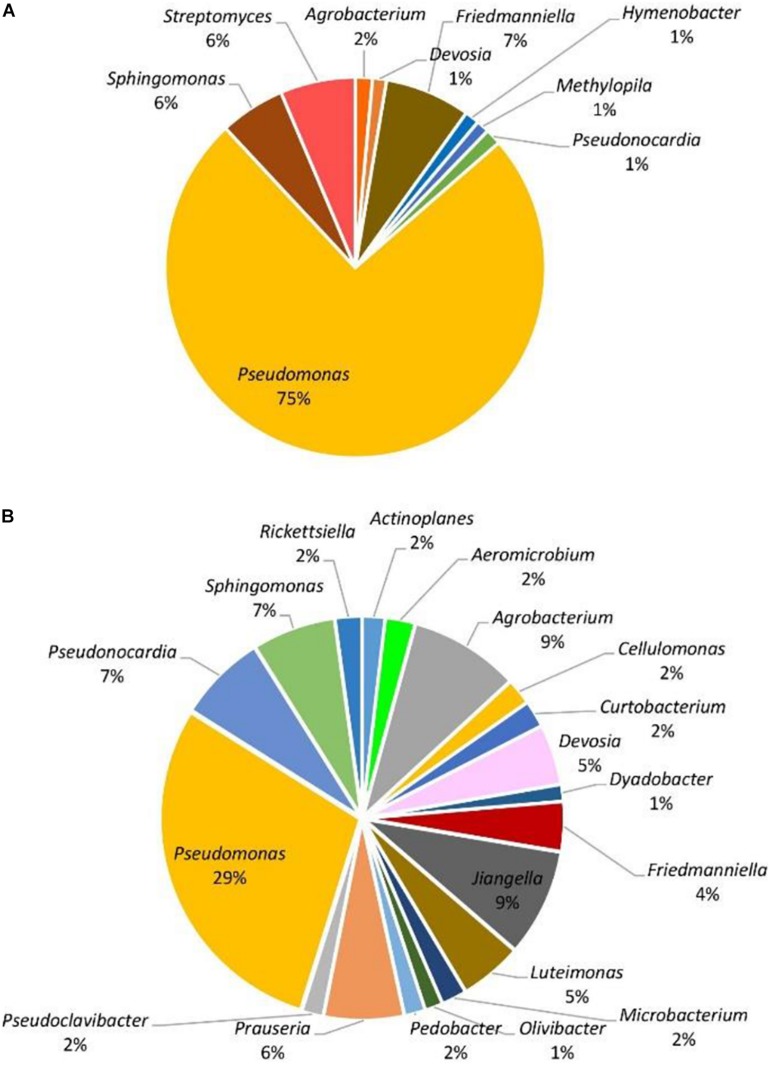
Genera of grapevine endophytic bacteria from Hilltops, NSW and their relative abundance to grapevines that were: **(A)** asymptomatic; and **(B)** symptomatic to grapevine trunk diseases.

**FIGURE 3:**
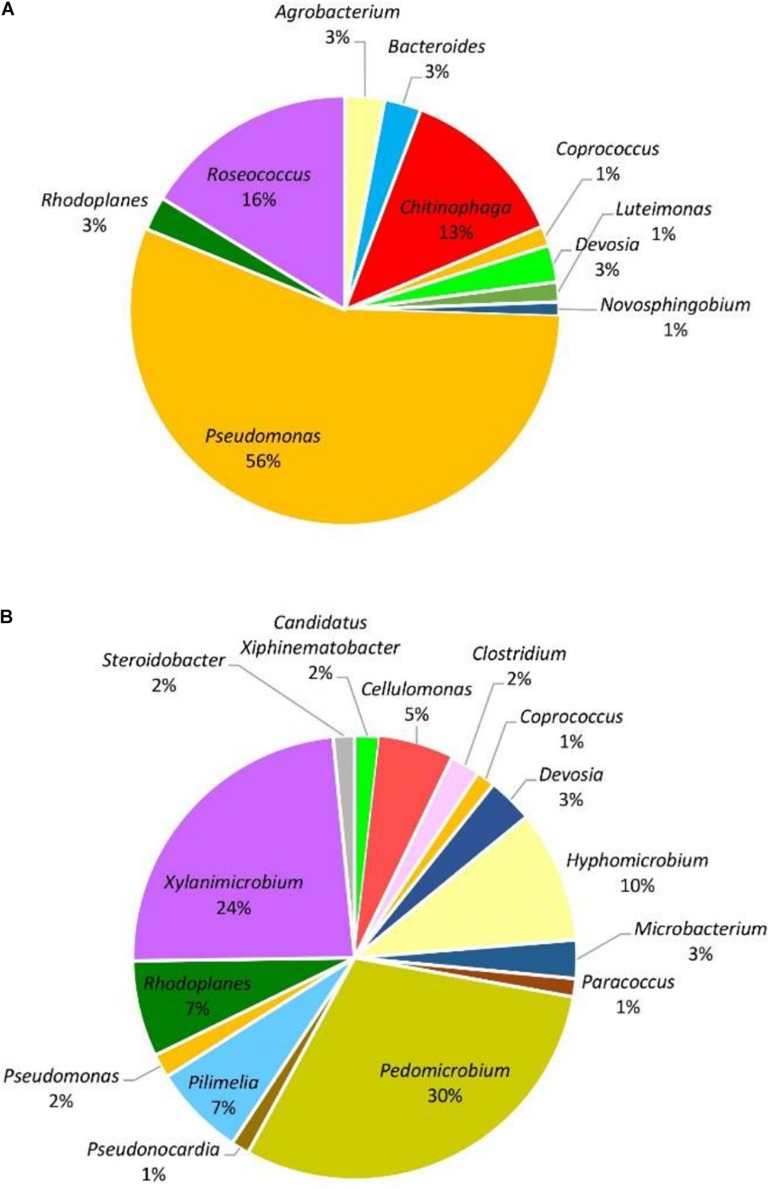
Genera of grapevine endophytic bacteria from the Hunter Valley, NSW and their relative abundance to grapevines that were: **(A)** asymptomatic; and **(B)** symptomatic to grapevine trunk diseases.

When considering abundance at the phylum and genus level, in all samples, the dominant phylum among the prokaryotic community was Proteobacteria, accounting for 51–96% of the total reads for the given taxa followed by Actinobacteria with 3–44%. The phyla Bacteroidetes, Firmicutes, and Chloroflexi were detected in less than 10% of the reads. At the genus level, with the exception of symptomatic samples from the Hunter Valley at 2% relative abundance, the most abundant genus within the phylum Proteobacteria was *Pseudomonas* with 75 and 29% in asymptomatic and symptomatic samples, respectively, from Hilltops, and 56% in asymptomatic vines from the Hunter Valley. It is also noteworthy that in both locations, *Pseudomonas* had higher relative abundance in samples asymptomatic for GTDs (Figures 2A, 3A) as compared to samples from symptomatic vines (Figures 2B, 3B). Symptomatic vines harbored higher diversity of the most abundant genera accounting for 19 and 15 bacterial genera from Hilltops and the Hunter Valley, respectively. In contrast, asymptomatic samples host a total of nine genera in Hilltops and 10 in the Hunter Valley. The genera *Pseudomonas, Sphingomonas, Agrobacterium, Deviosa, Friedmanniella*, and *Pseudonocardia* were present in both asymptomatic and symptomatic samples from Hilltops. The composition of bacterial genera from the Hunter Valley appeared to be more unique with only *Pseudomonas, Rhodoplanes, Coprococcus*, and *Deviosa* shared between asymptomatic and symptomatic samples. Other agriculturally important bacterial genera that occurred in greater than 1% relative abundance were *Streptomyces* and *Agrobacterium. Streptomyces*, which was detected in asymptomatic samples from Hilltops (6%), is implicated in plant disease control and plant growth promotion. *Agrobacterium*, which causes crown gall in grapevines, was identified in 1–9% of the total bacterial reads.

### Fungal Community

**FIGURE 4:**
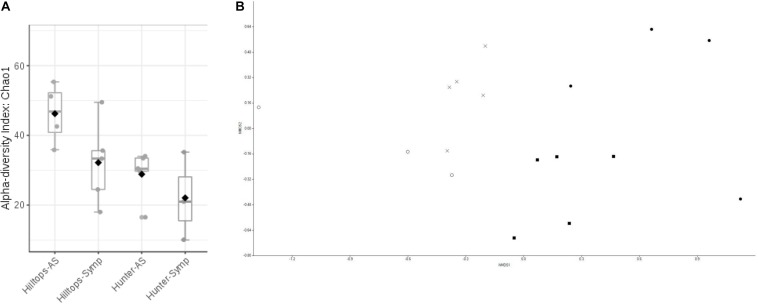
Diversity analysis of fungal endophytic communities sampled from GTD symptomatic and asymptomatic vines, in the Hilltops and Hunter Valley regions. Abundance of OTUs was determined following the sequencing of the ITS regions of rRNA genes: **(A)** Alpha-diversity measure using Chao1 at the OTU level, with each boxplot representing the diversity distribution of each factor (location and symptomology); and **(B)** Ordination based upon beta diversity analysis using the Bray Curtis index. Closed circles = Hilltops, asymptomatic; closed squares = Hilltops, symptomatic; X = hunter Valley, asymptomatic; O = Hunter Valley, symptomatic.

In terms of the fungal community, the richness of OTUs were significantly different across all factors (*F* = 1.3, *P* ≤ 0.05), being driven by location with Hilltops greater than the Hunter Valley (*t* = 2.12, *p* = 0.05; Figure 4A). When richness and evenness were considered, no significant differences at the OTU level were present. The compositional analysis of fungal communities, analyzed for beta diversity, indicated that while communities could be clustered, these were not strict and no significant differences were evident (*R* = 0.21, *P* > 0.05; Figure 4B).

**FIGURE 5:**
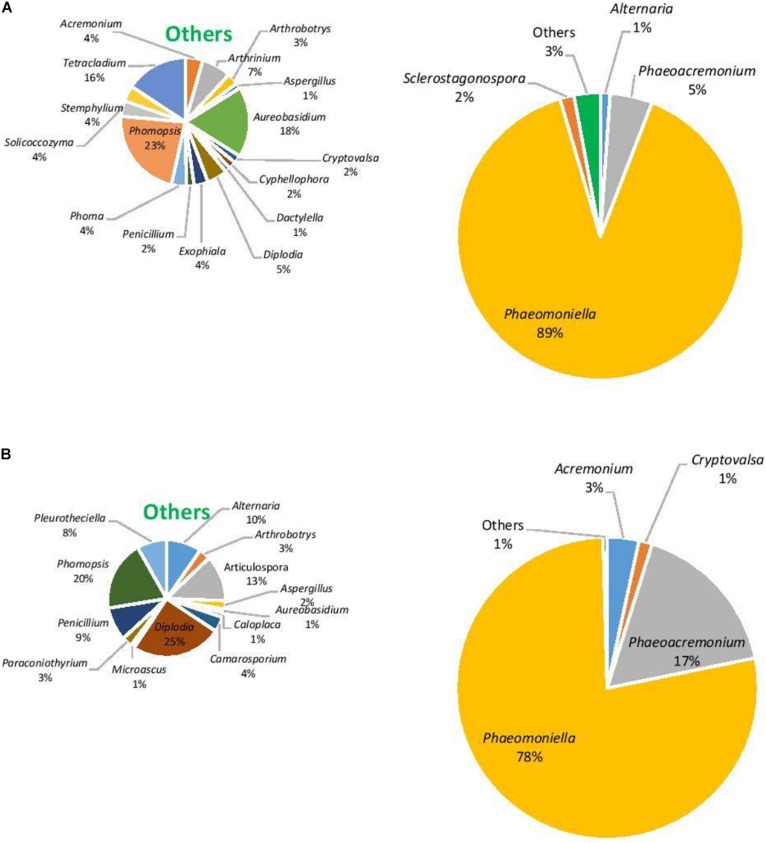
Genera of grapevine endophytic bacteria from Hilltops, NSW and their relative abundance to grapevines that were: **(A)** asymptomatic; and **(B)** symptomatic to grapevine trunk diseases.

**FIGURE 6:**
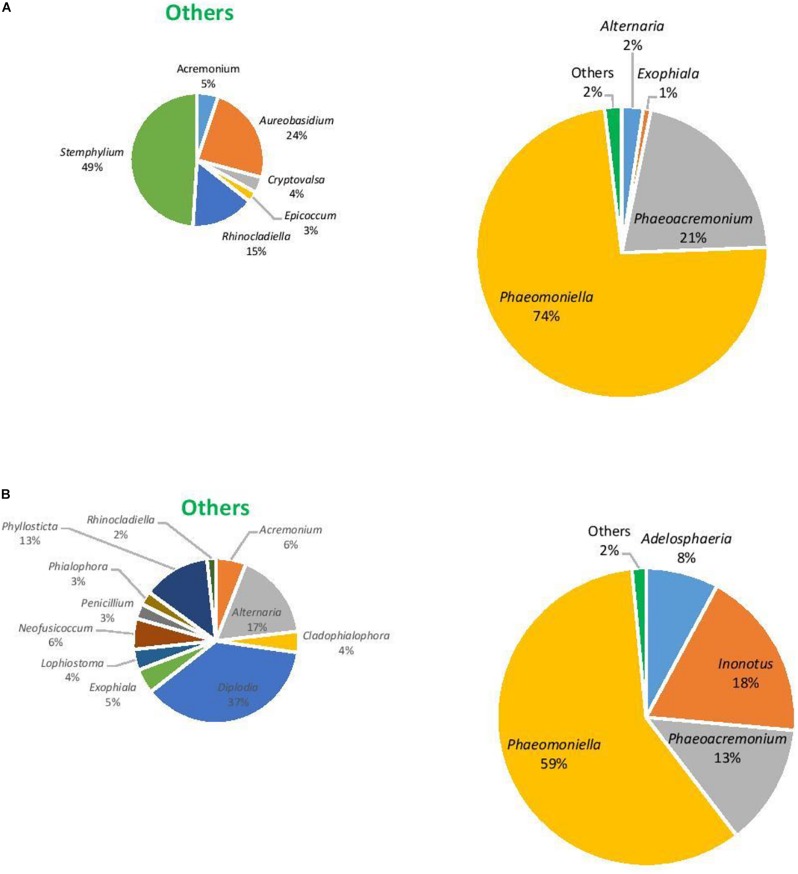
Genera of grapevine endophytic fungi from the Hunter Valley, NSW and their relative abundance to grapevines that were: **(A)** asymptomatic; and **(B)** symptomatic to grapevine trunk diseases.

The eukaryotic microbiome was mostly characterized by a preponderance of the phylum Ascomycota with greater than 99% relative abundance from both asymptomatic and symptomatic samples from Hilltops and asymptomatic tissues from the Hunter Valley. The relative abundance of Ascomycota in symptomatic vines from the Hunter Valley was 83% while phylum Basidiomycota was 17%. At the genus level, the fungal community was predominated by *Phaeomoniella* (primarily *P. chlamydospora*), representing 59–89% of the total fungi detected (Figures 5, 6). *Phaeomoniella* was more abundant in asymptomatic tissues accounting for 89 and 74% in Hilltops and the Hunter Valley (Figures 5A, 6A), respectively as compared to symptomatic tissues with 78 and 59% relative abundance (Figures 5B, 6B). This was followed by *Phaeoacremonium* species, specifically *Ph. iranianum* (5–21%) save for a divergence in asymptomatic vines from the Hunter Valley where *Inonotus* from the phylum Basidiomycota came in second at 18% relative abundance. *Phaeomoniella* and *Phaeoacremonium* species are GTD pathogens that are widely associated with Petri and Esca diseases of grapevines. *Inonotus* has also been isolated in some grapevines exhibiting Esca symptoms. Other trunk disease pathogens, *Cryptovalsa*, *Diplodia*, and *Neofusicoccum* were also detected in low abundance (0.01–1%). Some fungi implicated with various diseases of grapes and grapevines such as *Alternaria*, *Stemphylium*, *Phomopsis*, *Phyllostica*, *Penicillium*, *Phoma*, *Phialophora*, and *Aspergillus* were also noted in 0.01–2% of the eukaryotic population. Fungi with known biocontrol potential, including *Aureobasidium*, *Epicoccum*, and *Arthobotrys* were also found to inhabit the inner grapevine tissues.

### Isolation of Fluorescent *Pseudomonas* From Inner Grapevine Tissues

A total of 200 bacterial endophytes from Hilltops and the Hunter Valley samples and 10 bacterial strains from the CSU vineyard were isolated. From these, 57 isolates resembling *Pseudomonas* fluoresced under UV light at 365 nm when grown in King’s B medium. From among the 57 bacteria, 10 isolates exhibited antagonistic activity against the selected GTD pathogens (data not shown).

### *In vitro* Screening for Bacterial Endophytes Antagonistic Toward GTD Pathogens

#### Antagonism Against Botryosphaeria Dieback Pathogens

**FIGURE 7:**
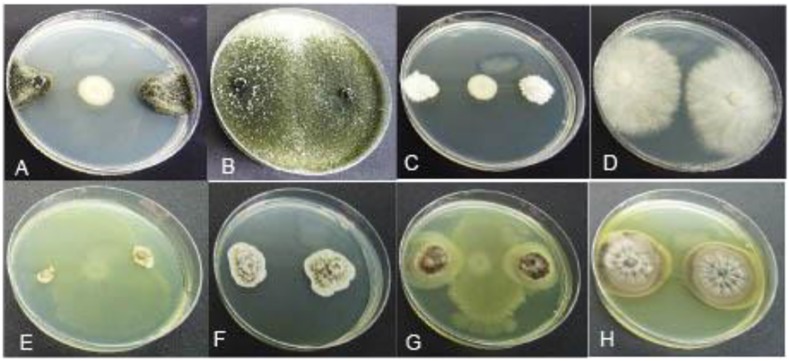
Mycelial growth inhibition of representative GTD pathogens by antagonistic *Pseudomonas* BCA strains: **(A)** *Dothiorella vidmadera* + BCA19, **(B)** *Do. vidmadera* only, **(C)** *Cryptovalsa ampelina* + BCA14, **(D)** *C. ampelina* only, **(E)** *Phaeomoniella chlamydospora* + BCA11, **(F)** *P. chlamydospora* only, **(G)** *Phaeoacremonium minimum* + BCA13, **(H)** *Ph. minimum* only.

The 10 *Pseudomonas* isolates showed varying degrees of antagonistic activity against Botryosphaeria dieback pathogens. Inhibition of mycelial growth was significantly affected by both BCAs and pathogens. Average mycelial inhibition caused by the different isolates of bacteria ranged from 12.0 to 43.1% with BCA11 and BCA13 having the highest inhibition (Table 4). BCA15 was the least inhibitory of the isolates examined. Sensitivity to BCAs was also significantly different (*P* ≤ 0.05) among the nine Botryosphaeria dieback pathogens that were tested, with *Dothiorella vidmadera* being the most sensitive (52.3% average) (Figures 7A,B) and *N. parvum* and *N. luteum* (15.4–18.5%) being the least sensitive.

**TABLE 4:** Mycelial growth inhibition of Botryosphaeria dieback pathogens by *Pseudomonas* strains as potential biocontrol agents (BCAs).

BCA	Percent mycelial growth inhibition (%)^d^									
	*BD*^a^	*DM*^a^	*DS*^a^	*DoV*^a^	*LT*^a^	*NA*^a^	*NL*^a^	*NP*^a^	*SV*^a^	BCA mean^b^
11	70.4 a	26.3abc	45.5 ab	66.5ab	41.0 a	46.8a	30.3a	20.4ab	40.9 b	43.1 A
12	58.1 a	0.0d	47.6 a	0.0d	38.5 a	0.0d	0.0c	10.1ab	62.0 a	24.0 F
13	68.9 a	25.9abc	37.6 abcd	53.5bc	44.4 a	36.0ab	29.5a	22.2a	58.1 a	41.7 A
14	64.1 a	29.4ab	39.2 abc	45.8c	23.5 b	37.8ab	25.0ab	21.0ab	42.7 b	36.5 B
15	12.6 b	14.1c	12.6 e	11.0d	11.5 b	12.3cd	12.0bc	8.5b	13.1 c	12.0 G
16	25.4 b	19.7bc	28.1 cd	69.4a	12.4 b	17.6c	14.2b	12.0ab	40.9 b	26.6 EF
17	19.5 b	24.1abc	25.5 de	72.8a	13.3 b	15.6c	14.9b	12.6ab	43.1 b	26.8 EF
18	22.9 b	29.2ab	29.5 cd	77.6a	13.8 b	17.8c	12.6bc	13.5ab	44.6 b	29.0 DE
19	22.9 b	35.5a	32.2 cd	79.1a	22.0 b	17.6c	20.8ab	13.1ab	45.0 b	32.0 CD
20	59.7 a	22.6abc	33.8 bcd	47.6c	24.0 b	32.4b	25.4a	21.0ab	38.5 b	33.9 BC
Pathogen mean^c^	42.3 W	22.7Y	33.1 X	52.3V	24.4 Y	23.4Y	18.5Z	15.4Z	42.9 W	

*^a^Means within a column followed by different letters are significantly different at P ≤ 0.05 LSD = 13.055 for BCA x pathogen interaction. ^b^BCA means within a column followed by different letters are significantly different at P ≤ 0.05 LSD = 4.352 for BCA means. ^c^Pathogen means within a row followed by different letters are significantly different at P ≤ 0.05 LSD = 4.128 for pathogen means. ^d^BD, Botryosphaeria dothidea; DM, Diplodia mutila; DS, D. seriata; DoV, Dothiorella vidmadera; LT, Lasiodiplodia theobromae; NA, Neofusicoccum australe; NL, N. luteum; NP, N. parvum; SV, Spencermartinsia viticola.*

#### Antagonism Against Eutypa Dieback Pathogens

All 10 *Pseudomonas* BCA isolates showed antagonistic activity against Eutypa dieback pathogens, however, mycelial growth inhibition differed significantly between bacterial strains and pathogens. Average mycelial inhibition caused by the different bacteria ranged from 8.3 to 64.6% with BCAs 11, 13, 14, 18, 19, and 20 having the greatest inhibition (Table 5). BCA15 was the least inhibitory among the BCAs. Sensitivity to BCAs was also significantly different (*P* ≤ 0.05) among the three Eutypa dieback pathogens that were tested, with *Cryptovalsa ampelina* (Figures 7C,D) and *Eutypella citricola* being more sensitive (53.8–51.0%) than *E. lata* (49.9%).

**TABLE 5:** Mycelial growth inhibition of the Eutypa dieback pathogens by *Pseudomonas* strains as potential biocontrol agents (BCAs).

BCA	Percent mycelial growth inhibition (%)			
	*Cryptovalsa ampelina*^a^	*Eutypa lata*^a^	*Eutypella citricola*^a^	BCA mean^b^
11	70.3ab	54.8abc	63.7 a	62.9AB
12	6.5d	49.8c	27.3 c	27.9D
13	70.6ab	50.9c	60.2 ab	60.6ABC
14	77.1a	51.8c	65.0 a	64.6A
15	6.2d	1.8d	16.8 d	8.3E
16	56.5c	53.4bc	55.4 ab	55.1C
17	59.3c	57.8abc	56.6 ab	57.9BC
18	61.6bc	62.6ab	56.0 ab	60.1ABC
19	59.3c	65.2a	53.1 b	59.2ABC
20	70.8ab	51.1c	55.0 ab	58.9ABC
Pathogen mean^c^	53.8X	49.9Y	51.0 XY	

*^a^Means within a column followed by different letters are significantly different at P ≤ 0.05 LSD = 10.462 for BCA × pathogen interaction. ^b^BCA means within a column followed by different letters are significantly different at P ≤ 0.05 LSD = 6.04 for BCA means. ^c^Pathogen means within a row followed by different letters are significantly different at P ≤ 0.05 LSD = 3.308.*

#### Antagonism Against Esca/Petri Disease Pathogens

**FIGURE 8:**
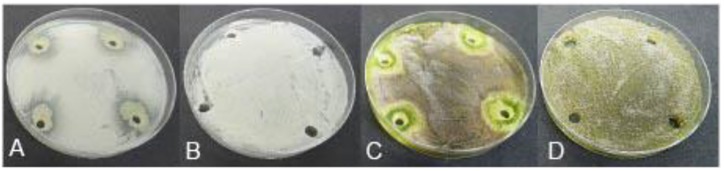
Antagonistic effect of *Pseudomonas* BCA strains on Esca/Petri disease pathogens: **(A)** *Phaeomoniella chlamydospora* + BCA17, **(B)** spores of *P. chlamydospora* only, **(C)** *Phaeoacremonium minimum* + BCA17, **(D)** spores of *Ph. minimum* only.

**FIGURE 9:**
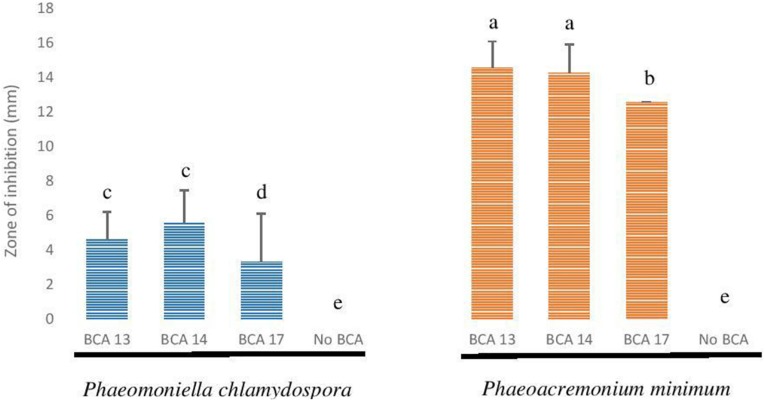
Antagonistic effect of selected *Pseudomonas* BCA strains on *Phaeomoniella chlamydospora* and *Phaeoacremonium minimum*.

The 10 *Pseudomonas* BCA isolates showed varying degrees of antagonistic activity against Esca/Petri disease pathogens. Mycelial growth inhibition was significantly affected by both BCAs and pathogens. Average mycelial inhibition caused by the different bacteria ranged from 9.7 to 44.9% with BCA11 and BCA13 having the highest inhibition (Table 6). BCA12 was the least inhibitory among the BCAs. Sensitivity to BCAs was also significantly different (*P* ≤ 0.05) between the Esca/Petri disease pathogens that were tested, with *P. chlamydospora* being more sensitive (55.0% average) (Figures 7E,F) than *Ph. minimum* (14.5%). However, although inhibition of mycelial growth by the BCAs was not as pronounced in *Ph. minimum*, an apparent change in the color along the edges of the hyphal colonies was observed (Figures 7G,H). Co-inoculation of *Ph. minimum* with the BCAs resulted in the disappearance of mycelial pigmentation along the margin indicating spore production was inhibited by the BCAs. Further tests on spore suspensions of *Ph. minimum* in a well dual culture assay showed the presence of zones of inhibition when cell suspensions of BCAs 13, 14, and 17 were present. Among the three BCAs, BCA11 produced the largest zone of inhibition at 21 mm (Figure 8). Average zones of inhibition produced by the three bacterial strains ranged from 19 to 21 mm and were significantly different from the control at *P* ≤ 0.05 (Figure 9).

**TABLE 6:** Mycelial growth inhibition of the Esca/Petri disease pathogens by *Pseudomonas* strains as potential biocontrol agents (BCAs).

BCA	Percent mycelial growth inhibition of Esca/Petri disease pathogens (%)		
	*Phaeomoniella chlamydospora*^a^	*Phaeoacremonium minimum*^a^	BCA mean^b^
11	70.3 a	17.3abc	43.8A
12	19.3 f	0.0d	9.7E
13	64.0 ab	25.8a	44.9A
14	53.7 cde	17.5*abc*	35.6*BCD*
15	60.7 abc	8.3cd	34.5*BCD*
16	59.7 bcd	12.6*bc*	36.1*BCD*
17	64.0 ab	13.6*bc*	38.8*ABC*
18	50.3 de	15.0*bc*	32.7C
19	47.7 e	15.2*bc*	31.5D
20	60.7 abc	19.3*ab*	40.0*AB*
Pathogen mean^c^	55.0 X	14.5y	

*^a^Means within a column followed by different letters are significantly different at P ≤ 0.05 LSD = 10.462 for BCA x pathogen interaction. ^b^BCA means within a column followed by different letters are significantly different at P ≤ 0.05 LSD = 6.604 for BCA means. ^c^Pathogen means within a row followed by different letters are significantly different at P ≤ 0.05 LSD = 3.308 for pathogen means.*

### Identification of Selected Bacteria by 16S rRNA Gene Sequencing

**FIGURE 10:**
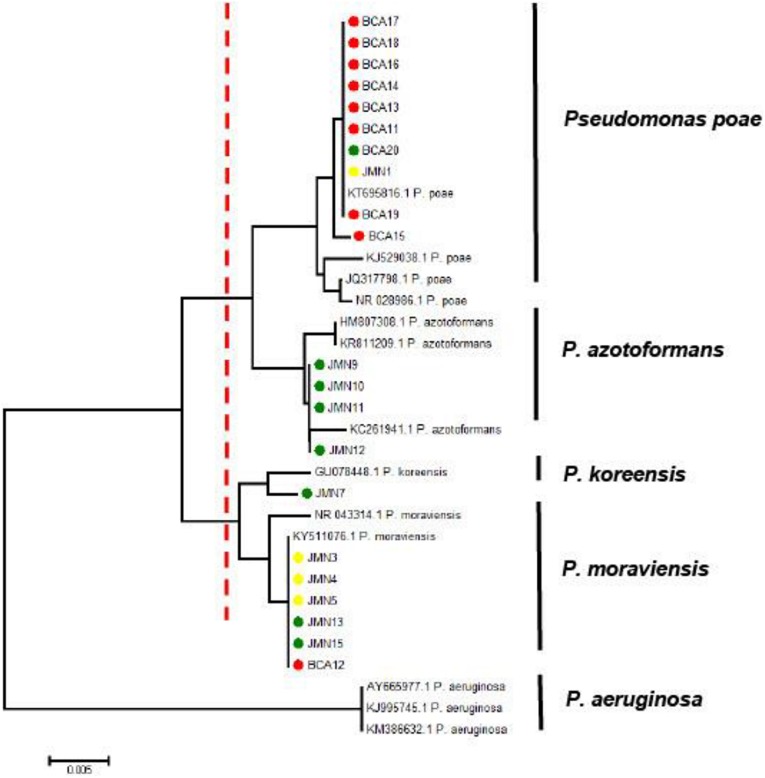
Neighbor joining tree obtained from the 16S rRNA gene sequence data of fluorescent bacteria isolated from inner grapevine tissues. The red dotted line indicates the main branches of the tree. Strains with red symbols are from the CSU vineyard, green from Hilltops, and yellow from the Hunter Valley. Reference sequences obtained from the NCBI data base are indicated by their accession numbers. The tree is rooted to the outgroup *P. aeruginosa.*

Querying the Genbank database with the 16S rRNA gene sequences generated from the 12 selected bacterial isolates, and those from the CSU vineyard, indicated these belonged to the genus *Pseudomonas.* Phylogenetic analysis of the aligned sequences from the bacterial strains and reference sequences obtained from Genbank resulted in a neighbor joining tree with two major branches (Figure 10). The first main branch was occupied by eight strains from CSU, five from Hilltops, and one from the Hunter Valley. This was further divided into two sub-branches. The first sub-branch consisted of eight strains from CSU and one strain each from Hilltops and the Hunter Valley, which clustered with *P. poae* reference strains. The second sub-branch was composed of four strains from Hilltops, which clustered with *P. azotoformans*. The second main branch was occupied by one strain from CSU, three from Hilltops, and three from the Hunter Valley and was divided into two sub-branches. The first sub-branch indicates a close relationship to *P. koreensis*. The second sub-branch is composed of one strain from CSU, two from Hilltops, and three from the Hunter Valley, which clustered with *P. moraviensis*. The fluorescent bacterial strains are all from the *P. fluorescens* complex (Garrido-Sanz et al., 2017). *P. aeruginosa* was included in the tree as an outgroup. Nine out of the 10 fluorescent bacterial strains (BCA11, BCA13–BCA20) that inhibited the mycelial growth of the GTD pathogens in the dual culture assay belong to the *P. poae* group. The remaining antagonistic strain, BCA12, is closely related to *P. moraviensis*.

## Discussion

This study investigated the diversity of the endomicrobiome associated with grapevines with and without symptoms of GTDs. Overall results showed that diversity of bacterial and fungal communities of grapevines with and without GTD symptoms differed between the regions examine, suggesting potential interactions between the beneficial and pathogenic organisms within a grapevine. To our knowledge, this study is the first attempt to investigate the microbiome of GTD symptomatic and asymptomatic wood tissues of grapevines in Australia, and the outcomes suggest that sampling of a number of agroecological zones would be beneficial to improve our understanding of endophytic communities. The microbial diversity data revealed a preponderance of the genus *Pseudomonas* in grapevine tissues that were asymptomatic to GTD in both locations, accounting for 56–75% relative abundance and in symptomatic tissues from Hilltops with 29% abundance. This concurs with the results of Bell et al. (1995), Campisano et al. (2015), Faist et al. (2016), and Deyett et al. (2017) who reported *Pseudomonas* to dominate the bacterial assemblage in grapevines in separate studies conducted in California, Germany, Italy, and Canada, respectively. Interestingly, in a study conducted in Australia, West et al. (2010) reported *Bacillus* to be the most frequently isolated bacterium in grapevine canes, comprising 55% of the total. *Pseudomonas* spp., *Curtobacterium* spp., *Burkholderia* spp., and *Streptomyces* spp. accounted for 34% of the total endophytic bacteria isolated. *Bacillus* was not detected in the current samples even though these were also collected from grapevines in New South Wales, Australia. The difference in the microbial composition may be explained by the difference in the age of the vines as West et al. (2010) sampled from relatively younger vines (10 years old) as compared to the ≥20 years old grapevines sampled in this study. Similar observations were reported by Bruez et al. (2015) where *Bacillus* sp. and *Pantoea agglomerans* were the most commonly isolated bacteria from 12 years old grapevine wood samples either symptomatic or asymptomatic for esca-foliar symptoms. Andreolli et al. (2016) identified age of the vine as an important factor affecting the composition of bacterial endophytes in grapevines, as well as genus richness, with greater genus richness observed in younger vines. The results obtained in this current research indicated higher diversity of bacterial genera in symptomatic vines, hosting 15–19 genera as compared with 9–10 for asymptomatic vines. While these were not found to be significant differences, these were used to provide the indicator for identifying organisms contributing to the effect. Other factors may also influence the composition of the grapevine microbiome. Campisano et al. (2015) found bacterial endophytic communities to be affected by the pest management strategy employed in the vineyard, and revealed differences in microbial composition in grapevines cultivated using organic production as opposed to those using integrated pest management (IPM). The health status of the vine (Bulgari et al., 2011; Bruez et al., 2014; Faist et al., 2016), the growing region, cultivar and climate (Bokulich et al., 2013; Bruez et al., 2014; Portillo et al., 2016) may also shape the community of microorganisms within the grapevine. The results presented in this current research concur with past findings as location was the influencing factor for differences in endophytic diversity.

Higher relative abundance of *Pseudomonas* in asymptomatic tissues observed in this study may impart a suppressive effect on GTD pathogens, and suggests a plausible antagonistic role of *Pseudomonas* in inhibiting symptoms of GTDs. In a similar study, Deyett et al. (2017) found a decrease in the relative abundance of *Xylella fastidiosa*, a xylem-limited bacterium causing Pierce’s disease in grapevines, in the presence of *Achomobacter xylosoxidans* and *P. fluorescens*. The presence of grapevines asymptomatic for Pierce’s disease in vineyards with high disease pressure was attributed to the microbial community that inhabits the grapevine vascular endosphere.

This study also showed that the eukaryotic community was dominated by the genus *Phaeomoniella* in both asymptomatic and symptomatic grapevines, representing 74–89% and 59–78% of the total fungi, respectively. *Phaeoacremonium* came in second, with 5–21% relative abundance except in asymptomatic vines from the Hunter Valley where *Inonotus* had a higher relative abundance at 18%. *P. chlamydospora* and *Ph. aleophilum* (syn. *Ph. minimum*) have been implicated in two GTDs: Petri disease (previously known as black goo decline) and Esca (Crous and Gams, 2000; Mostert et al., 2006b). *Inonotus* has also been reported in grapevines exhibiting white heart rot, a symptom that is also generally associated with Esca (Edwards and Pascoe, 2004; Fischer et al., 2005; González et al., 2009; White et al., 2011). Occurrence of these three dominant fungi in grapevines showing symptoms of Petri disease and Esca has previously been reported in Australian vineyards. Petri disease was found to be expressed in 82% of the diseased samples (from a total of 124 symptomatic samples) while only 18% were diagnosed with Esca (Edwards and Pascoe, 2004). Moreover, *P. chlamydospora* was found to be widely distributed in Australia and was detected in Western Australia, South Australia and Victoria in 98% of the samples while *Ph. minimum* was isolated in only 15% of the samples. The current fungal diversity profile indicates *P. chlamydospora* to be the dominant fungal species but *Phaeoacremonium* is from the species *Ph. iranianum* in contrast to *Ph. minimum* that was previously reported in Australia. *Ph. iranianum*, which has been reported to be isolated from *Vitis vinifera* in Iran, Italy and South Africa (Mostert et al., 2006a; White et al., 2011) is phylogenetically and morphologically close to *Ph. minimum* and can only be differentiated by the predominance of type III phialides and by its subcylindrical type II phialides (Mostert et al., 2006a). Its presence indicated here by diversity profiling would need to be confirmed by these conventional techniques.

The diversity profiling revealed a higher abundance of *Phaeomoniella* in asymptomatic grapevines than in the symptomatic tissues. In France, Bruez et al. (2016) was able to recover the Esca pathogens, *Ph. minimum* and *P. chlamydospora* and the white rot fungi, *Fomitiporia mediterranea* and *Stereum hirsutum*, consisting 50% of all the fungal taxa isolated, from 42 years old vines that did not express symptoms of grapevine trunk disease. Edwards and Pascoe (2004) were also able to isolate *P. chlamydospora* in symptomless grapevines, as did Mugnai et al. (1999). They postulated that the fungus may initially behave as an endophyte or latent pathogen, only becoming pathogenic when the grapevines are stressed, after which Petri disease starts to manifest. Furthermore, symptoms of Esca are erratic and may not show every year (Edwards et al., 2001). Hofstetter et al. (2012) also found the same taxa of presumed Esca-associated fungal pathogens, including *P. chlamydospora* and *Phaeoacremonium* spp., in both diseased and healthy grapevines that were 15–30 years old. This indicates that these fungi are part of the normal mycota associated with adult grapevines. Ferreira et al. (1999) reported water stress to be a predisposing factor for *P. chlamydospora* infection and slow dieback of grapevines in South Africa. Plant genotype (Marchi, 2001), age (Mugnai et al., 1999), and climate (van Niekerk et al., 2011) are also factors that may affect the susceptibility of the grapevines to the disease. Environmental changes may also elicit various plant responses (i.e., development of thicker wax layers on leaves or changes in stomatal densities) that could affect infection and expression of symptoms (Fischer and Peighami Ashnaei, 2019).

Abundance of the *Pseudomonas* spp. in asymptomatic grapevines led to the further investigation of the antagonistic activity of these bacteria against GTDs. The results of the dual culture assays revealed 10 strains of grapevine endophytic *Pseudomonas* with antagonistic activity against Botryosphaeria dieback, Eutypa dieback and Esca/Petri disease pathogens based on their ability to inhibit mycelial growth in culture. *Pseudomonas* spp. also appeared to affect spore production of *Ph. minimum* as demonstrated by the presence of a zone of inhibition between the bacterium and the spores of the pathogen in a well dual culture assay. This suggests that the *Pseudomonas* spp. are capable of producing potent antifungal agents that leached into the agar and inhibited the growth of the fungal pathogens. Of the *Pseudomonas* strains identified, nine out of the 10 were closely related to *P. poae*. *P. poae*, originally isolated from the phyllosphere of grasses, has been reported as an endorhiza of sugar beet (Behendt et al., 2003; Müller et al., 2013; Xia et al., 2019). It is used as a BCA and is applied as a seed treatment to suppress late root rot in sugar beet (Zachow et al., 2010). The other antagonistic bacterium, BCA12, belongs to the same clade as *P. moraviensis* which has been reported as a soil bacterium implicated in the suppression of Rhizoctonia diseases of potato (Tvrzová et al., 2006; Mrabet et al., 2013; Miller et al., 2016). However, the *in vitro* assay conducted in the current study indicated that BCA12 was less inhibitory compared to the other nine BCAs from the *P. poae* group.

Although *P. poae* has been identified to have biocontrol potential, the exact mechanism of control is yet to be elucidated. Unlike other species of *Pseudomonas* that are more popularly known to produce 2,4 diacetylphloroglucinol, phenazine 1-carboxylic acid and its derivatives, and the siderophores pyoluteorin and pyrrolnitrin, which are antifungal metabolites identified with *Pseudomonas*, *P. poae* lacks the ability to produce these compounds (Mavrodi et al., 2012). Instead, it was found to be a good rhizosphere colonizer with exoprotease activity (Mavrodi et al., 2012). Niem et al. (2019) identified putative biocontrol gene clusters in the genomes of BCAs 13, 14, and 17 (*P. poae*) responsible for the biosynthesis of the secondary metabolites Poaeamide, Rhizomide and Rhizoxins. Gene clusters for production of lipopeptide antibiotics, siderophores, proteases, detoxification, lipopolysaccharide, multidrug resistance, microbe-associated molecular proteins (MAMPS), and biofilms were likewise detected (Niem et al., 2019). Production of other antifungal compounds, possibly not previously reported in other *Pseudomonas* species, requires further investigation. Furthermore, the efficacy of *P. poae* to suppress trunk disease infection *in planta* should be investigated to ascertain its efficacy as a biocontrol agent.

The results of this study indicate that some grapevine endophytic bacteria such as *P. poae* may be potential BCAs which could be used to control GTD pathogens. Further tests are currently being conducted to assess the bioactivity of these BCAs *in planta*.

## Data Availability Statement

The datasets generated for this study can be found in the Genbank Accession numbers: MN480480-MN480489. Raw sequence data for the diversity profiling is available through BioProject PRJNA605898.

## Author Contributions

JN conducted the research, analyzed the data, and wrote the manuscript. RB-B, SS, and BS contributed to the direction of the research and advised on analysis and interpretation of the data. All co-authors revised the manuscript.

## Conflict of Interest

The authors declare that the research was conducted in the absence of any commercial or financial relationships that could be construed as a potential conflict of interest.

## Footnotes

1 www.ncbi.nlm.nih.gov
